# Case Report: Chemotherapy Indication in a Case of Neurofibromatosis Type 1 Presenting Optic Pathway Glioma: A One-Year Clinical Case Study Using Differential Tractography Approach

**DOI:** 10.3389/fnhum.2021.620439

**Published:** 2021-04-06

**Authors:** Amir Mohammad Pajavand, Guive Sharifi, Amir Anvari, Farahnaz Bidari-Zerehpoosh, Mohammad A. Shamsi, Saeedeh Nateghinia, Tohid Emami Meybodi

**Affiliations:** ^1^Institute for Cognitive and Brain Sciences, Shahid Beheshti University Government College University, Tehran, Iran; ^2^Skull Base Research Center, Loghman Hakim Hospital, Shahid Beheshti University of Medical Sciences, Tehran, Iran; ^3^Department of Radiation Oncology, Shahid Beheshti University of Medical Sciences, Tehran, Iran; ^4^Department of Pathology, Loghman Hakim Hospital, Shahid Beheshti University of Medical Sciences, Tehran, Iran; ^5^Functional Neurosurgery Research Center, Shohada Tajrish Hospital, Shahid Beheshti University of Medical Sciences, Tehran, Iran

**Keywords:** neurofibromatosis type 1, diffusion tensor imaging, optic pathway glioma, immunohistochemistry, short-wavelength automated perimetry, temozolomide, low-grade glioma, visual field index

## Abstract

Neurofibromatosis type 1 (NF1) is associated with peripheral and central nervous system tumors. It is noteworthy that the regions in which these tumors frequently arise are the optic pathways (OPs) and the brainstem. Thus, we decided to trace the procedure of diffusion Magnetic Resonance Imaging (dMRI) alterations along with Short-Wavelength Automated Perimetry (SWAP) examinations of the OPs after surgery and chemotherapy over 1 year, which enabled us to evaluate chemotherapy's efficacy in an NF1 patient with an OP tumor. In this study, a 25-year-old woman with NF1 and left optic radiation (OR) glioma underwent surgery to remove the glioma. Immunohistochemistry (IHC) revealed a Pilocytic Astrocytoma (PA) WHO grade I. Post-operation chemotherapy done using nine treatment cycles of administering Temozolomide (TMZ) for 5 days every 4 weeks. Applying the region of interest (ROI) differential tractography method and SWAP four times every 3 months allowed us to follow the patient's visual acuity alterations longitudinally. The differential deterministic tractography method and statistical analyses enabled us to discover the white matter (WM) tracts anisotropy alterations over time. Furthermore, statistical analyses on the SWAP results along time illustrated possible alterations in visual acuity. Then, we could compare and associate the findings with the SWAP examinations and patient symptoms longitudinally. Statistical analyses of SWAP tests revealed a significant improvement in visual fields, and longitudinal differential tractography showed myelination and dense axonal packing in the left OR after 1 year of treatment. In this study, we examined an old hypothesis suggesting that chemotherapy is more effective than radiotherapy for NF1 patients with OP gliomas (OPGs) because of the radiation side effects on the visual field, cognition, and cerebrovascular complications. Our longitudinal clinical case study involving dMRI and SWAP on a single NF1-OPG patient showed that chemotherapy did not suppress the OP myelination over time. However, it should be noted that this is a clinical case study, and, therefore, the generalization of results is limited. Future investigations might focus on genetic-based imaging, particularly in more cases. Further, meta-analyses are recommended for giving a proper Field Of View (FOV) to researchers as a subtle clue regarding precision medicine.

## Introduction

The incidence of NF1 as a tumor suppressor syndrome has occurred in ~1 out of every 2500–3000 people worldwide (Huson et al., 1988; Compston, 1994; Hughes, 1999; Margaret, 2000). NF1 is associated with peripheral and central nervous system tumors. Most NF1 tumors occur in children, and most of these tumors arise within the OPs and hypothalamus (Korf, 2000; Albers and Gutmann, 2009).

The most frequent tumors associated with NF1 are PAs, WHO grade I tumors (Szudek et al., 2000); this is supported by other studies in the past decade, wherein 100 tumor patients with NF1 were evaluated in 2008, and 49% of the cases under 20 years old revealed PA tumors, while only 27% of adults exhibited other tumors (WHO grade II, III, and IV) (Rodriguez et al., 2008). Furthermore, in another study of 23 high-grade and 32 low-grade gliomas in NF1 patients, 77% of pediatric gliomas were low-grade while 78% of adult tumors were high-grade (D'Angelo et al., 2019).

Following the literature, the surgical resection of NF1 glioma tumors is not feasible due to their location and vision loss or hydrocephalus (Listernick et al., 2007). In addition, radiation therapy might lead to Moyamoya syndrome in NF1 patients (Ullrich et al., 2007). Also, radiotherapy in NF1 OPGs correlated with poor visual outcomes, increased mortality (Sievert et al., 2013), and the occurrence of secondary brain tumors and vasculopathy (Sharif et al., 2006; Merchant et al., 2009; Helfferich et al., 2016).

As a consequence of these risks, chemotherapy is considered an alternative approach for NF1 OPGs characterized by diminishing visual acuity. In this respect, chemotherapeutic agents used in the treatment include vincristine, carboplatin, vinblastine, vinorelbine, and TMZ (Packer et al., 1997; Bouffet et al., 2012; Cappellano et al., 2015). However, the sue of chemotherapeutic agents barely recovers any premorbid visual acuity (Dalla Via et al., 2007; Kalin-Hajdu et al., 2014).

In this study, a 25-year-old young adult woman with a left OR PA was studied longitudinally. According to the literature, the patient is considered a rare NF1-OPG patient. Because of the involvement of the left OR's large glioma with its massive edema, surgery was performed, and the tumor resected. We decided to utilize previously proposed hypotheses and experiences for the post-operation strategy, which led us to implement chemotherapy instead of radiation to avoid visual loss, cognitive problems, and vasculopathy.

To evaluate this hypothesis, we decided to use longitudinal DTI connectometry and SWAP examinations for 1 year. Differential statistical analyses were also performed to assess the outcomes of this investigation.

## Materials and Methods

### Medical History and Treatment Strategy

**Figure 1:**
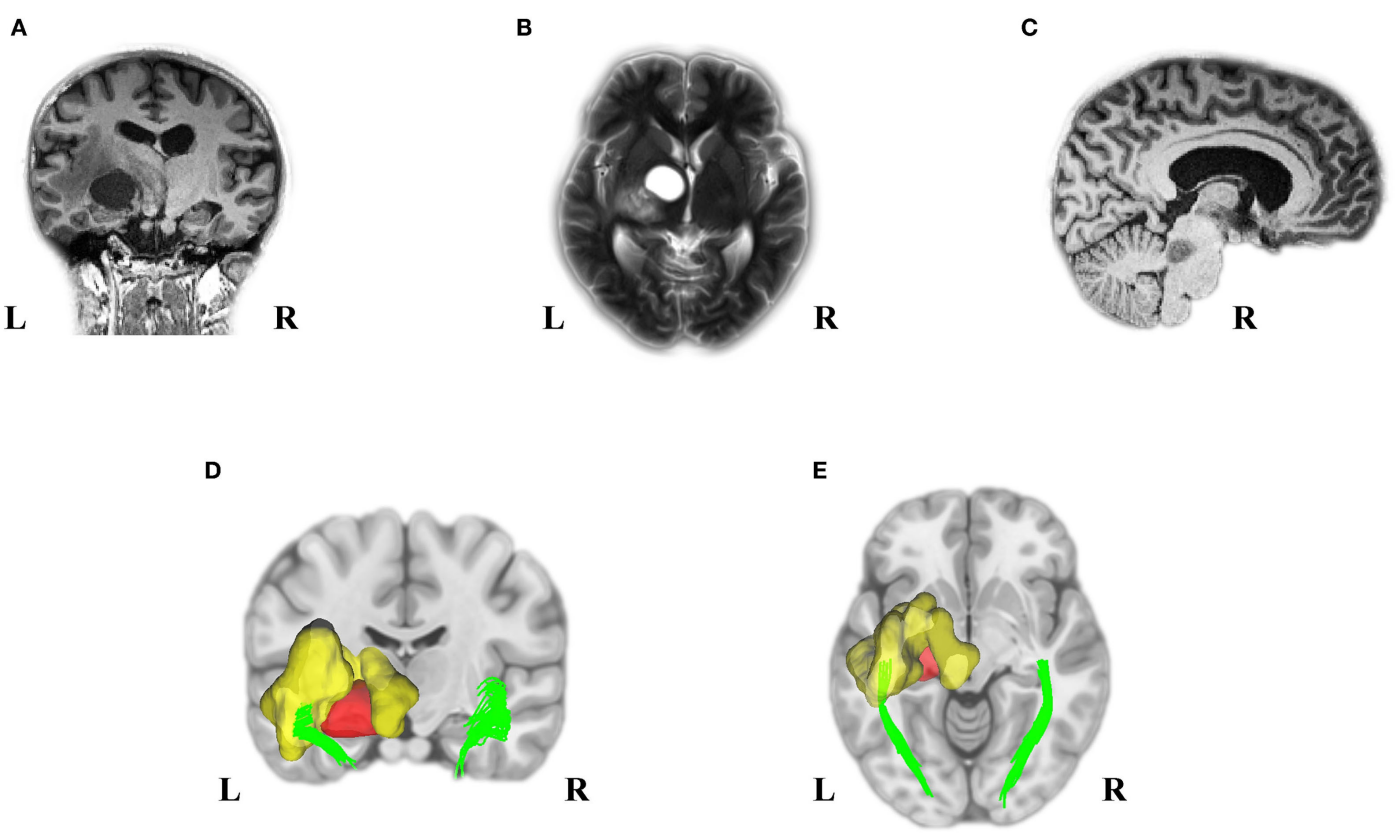
Pre-Operation MRI and analyzed DTI scans displayed multiple tumors in our young adult NF1 patient. It is not uncommon for NF1 patients to have more than one CNS tumor. As the MRI presented, the NF1 brain showed a left OR glioma **(A)**, Left thalamus cyst **(B)**, and a right midbrain tumor **(C)**. Deterministic tractography demonstrated the left OR involvement because of the left WM OR glioma with its massive edema **(D,E)**. MRI, magnetic resonance imaging; DTI, diffusion tensor imaging; NF1, neurofibromatosis type 1; CNS, central nervous system; OR, optic radiation; WM, white matter.

A 25-year-old woman was referred to the hospital due to visual impairment. The MRI scan revealed multiple lesions, including a left Thalamus cyst, a right lesion of the pons, and a WM OR glioma with massive edema (see Figure 1).

Clinical diagnosis requires at least two of seven criteria (Gutmann, 1988; Gutmann et al., 1997) to confirm the presence of NF1.

Our patient presented with significant signs of several criteria, including the presence of eight or more café au lait macules (>1.5 cm) (see Supplementary Figures 1A,B), the presence of 15 or more cutaneous/subcutaneous neurofibromas (see Supplementary Figures 1A,B), axillary freckling (see Supplementary Figure 1C), and OP glioma (see Supplementary Figure 1D). The patient did not exhibit any oral manifestation of the ailment and demonstrated no balance problems or scoliosis.

Usually, after surgery, LGG patients receive systemic chemotherapy, such as PCV (Procarbazine, CCNU, and Vincristine) or TMZ. PCV and TMZ provide similar response rates (45–62%) and durations (10–24 months), with the toxicity profile favoring temozolomide in terms of tolerability (reduced myelotoxicity) (Quinn et al., 2003; Soffietti et al., 2010).

This idea has extended to national guidelines that list single-agent TMZ and multi-agent PCV as equally appropriate options (Ziu et al., 2015). Based on the available evidence and Duke University's clinical trial (NCT00003466^1^), we chose a single-agent TMZ agent for post-surgery chemotherapy surgery. Thus, the patient received TMZ orally (200 mg/m^2^ per day) for 5 days every 4 weeks. The patient also received nine treatment cycles for the PA WHO grade I as a progressive LGG.

## Assessments

### Short-Wavelength Automated Perimetry (SWAP)

**Figure 2:**
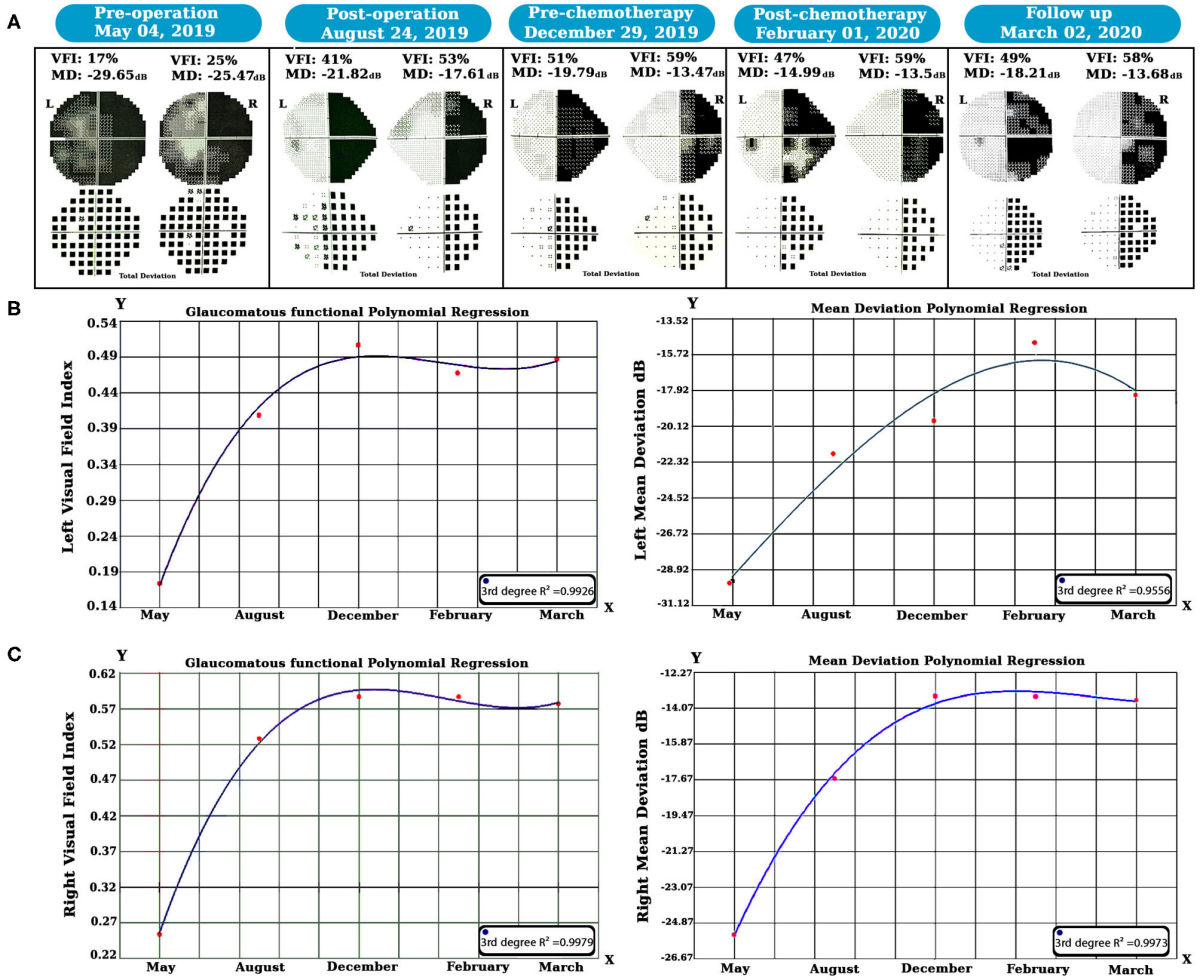
Longitudinal SWAP examinations and statistical analyses were done. Perimetric tests revealed left visual field healing, but a homonymous hemianopsia defect occurred due to the left geniculate WM glioma **(A)**. The polynomial regression method showed that both side vision acuity improved over time **(B,C)**. SWAP, Short-Wavelength Automated Perimetry; WM, white matter.

SWAP is a visual field examination designed to assess the short-wavelength-sensitive color system (Drance et al., 1981; Flammer and Drance, 1984). According to our strategy, we decided to utilize SWAP four times every 3 months for 1 year to examine the visual field. The data from each SWAP test were extracted and analyzed (see Figure 2) via the polynomial regression method (Ostertagováa, 2012).

### Structural and Microstructural MRI Data Acquisition

#### DTI Data Acquisition

All structural MRI scans were acquired from 3T MRI scanners (Siemens Prisma). 3D T1 MPRAGE anatomic acquisitions were made [1 mm slice, 256 × 256 matrices, echo time (TE) = 3.74 ms, repetition time (TR) = 1,810 ms, flip angle = 30°] and used to superimpose DTI images. A diffusion-weighted imaging (DWI) brain scan was also done with the same scanner with a 64-channel head coil. Other acquisition parameters were as follows: number of slices, 68; diffusion directions, 30; FOV, 256 × 256 mm^2^; voxel size, 2 × 2 × 2 mm^3^; TR/TE, 9,000/90 ms.

#### DTI Data Processing

To preprocess the DTI data, we utilized the FMRIB Software Library, FSL 6.0 (Woolrich et al., 2009; Jenkinson et al., 2012). At first, all diffusion-weighted images were checked visually for any visible artifacts and then corrected for B0 inhomogeneities and eddy-current distortion. Each subject's DWI was registered to the corresponding b = 0 images via affine transformation.

Second, the case's DWI data were reconstructed via Q-Space Diffeomorphic Reconstruction (QSDR), a method for calculating the directional distribution of the water diffusion density in standard space using DSI Studio software (Yeh and Tseng, 2011; Yeh et al., 2013).

To perform ROI-based individual connectometry, three normal age- and sex-matched individuals were added to the study for the analyses. Notably, just three structural MRI and dMRI scans were collected for our research, and no longitudinal data acquisition was performed for the normal subjects.

### Statistical Analyses

#### Differential Tractography

We used differential tractography to track the anisotropy difference along WM tracts over time. This technique was developed to compare the dMRI scans of a single patient at different times by mapping the DTI data into a common space and tracing the connectivity alterations through all or specific WM fiber tracts (Yeh et al., 2019). By focusing on the anisotropy difference, we can examine the tracking's stability across two periods.

The initial tracts for differential tractography were generated to create a series of 100,000 seeds with the same initial tracking parameters. We then used quantitative anisotropy (QA) instead of normalized quantitative anisotropy (NQA) to extract the connectivity percent change based on QA.

We determined that the initial QA threshold was 10% of the actual QA values. We looked at the QA changes with three different thresholds (+10%, +20%, and +30%) to make connectivity comparisons between time points.

#### False Discovery Rate Estimation

Per our strategy, the patient underwent dMRI scans four times every 3 months. We used this method to illustrate the WM tracts with increased anisotropy. Supplementary Figure 2 demonstrates our experimental design, wherein true-positive and false-positive findings were obtained that enabled us to estimate the false discovery rate (FDR).

To estimate the false-positive findings using this approach, we needed to obtain a sham scan on the same day as the baseline scan. Any positive results from the sham scan would be considered false-positive findings.

Then, the sham scan was compared with the baseline scan. As presented in Supplementary Table 1, the false and true positive findings were estimated, and the FDR was calculated. We then used our dataset to find the pre-chemotherapy and post-chemotherapy dMRI changes. We used the pre-chemotherapy scan as the baseline and the post-chemotherapy scan as the study scan; the sham scan was also included. Moreover, to evaluate the pre-operation and post-chemotherapy DTI changes, the same method was used.

#### Diffusion Metric Alterations Overtime and Illustration

**Figure 3:**
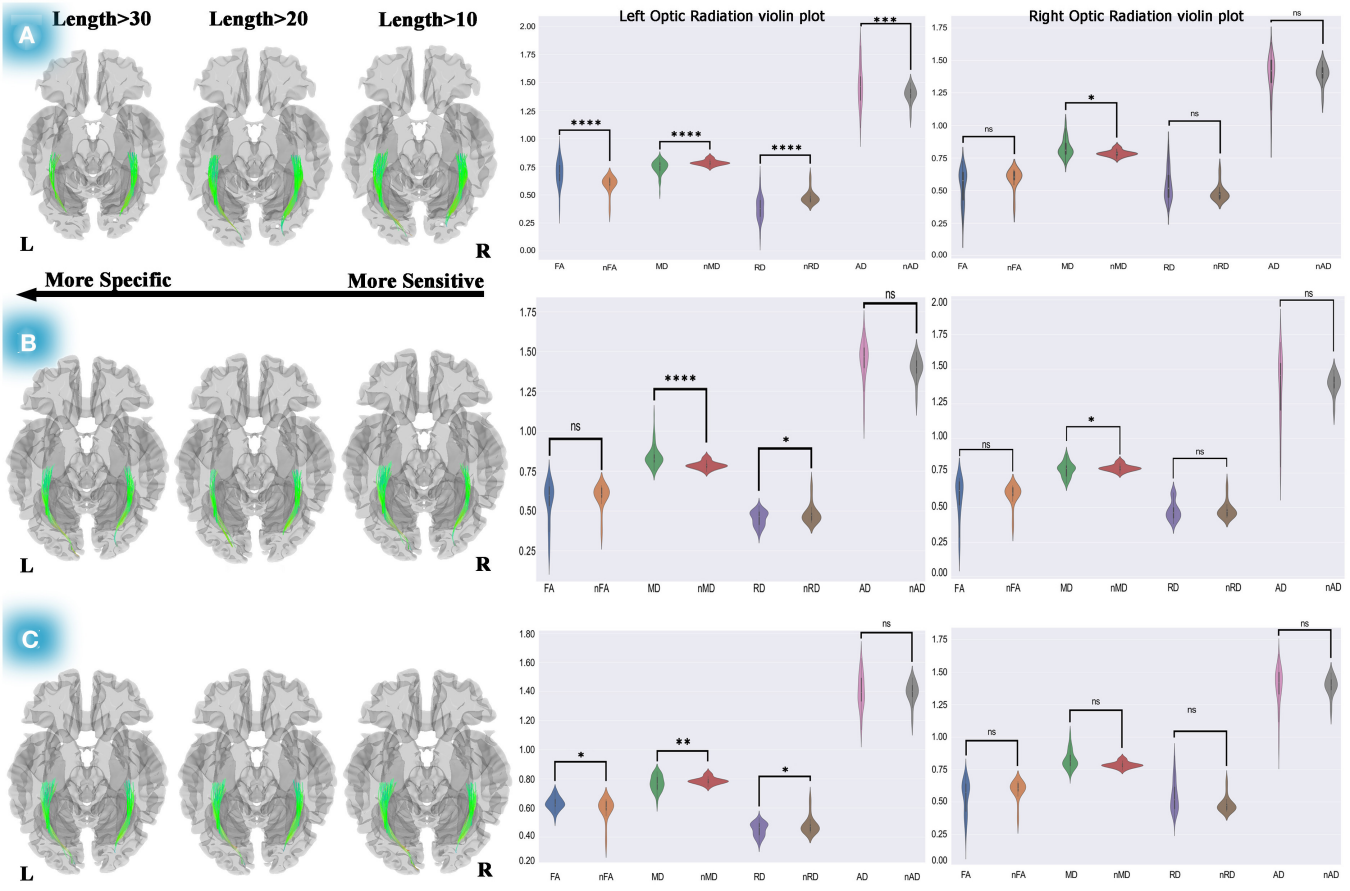
Differential tractography approach and statistical analyses to discover the ORs connectivity and diffusion metric alterations. The analyses on pre-chemotherapy compared with pre-operation showed increased connectivity in the left and right OR. Furthermore, violin plots showed fiber maturation according to increased FA, decreased MD, and RD **(A)**. This method used for post-chemotherapy vs. pre-chemotherapy revealed increased connectivity in the left and right OR, and statistical analyses on diffusion metrics showed no pathological indication **(B)**. This method also revealed increased connectivity of both ORs after 1-year of post-operation compared to the pre-operation, and statistical analyses presented increased FA, decreased MD, and RD, which could be a picture of myelination and dense axonal packing in the left OR **(C)**. Statistics performed on the diffusion metrics by two-tailed sample *t*-test, *P < 0.05; **P < 0.01; ***P < 0.001; ****P < 0.0001. OR, optic radiation; FA, fractional anisotropy; MD, mean diffusivity; RD, radial diffusivity; AD, axial diffusivity; nFA, normal FA; nMD, normal MD; nRD, normal RD; nAD, normal AD.

**Figure 4:**
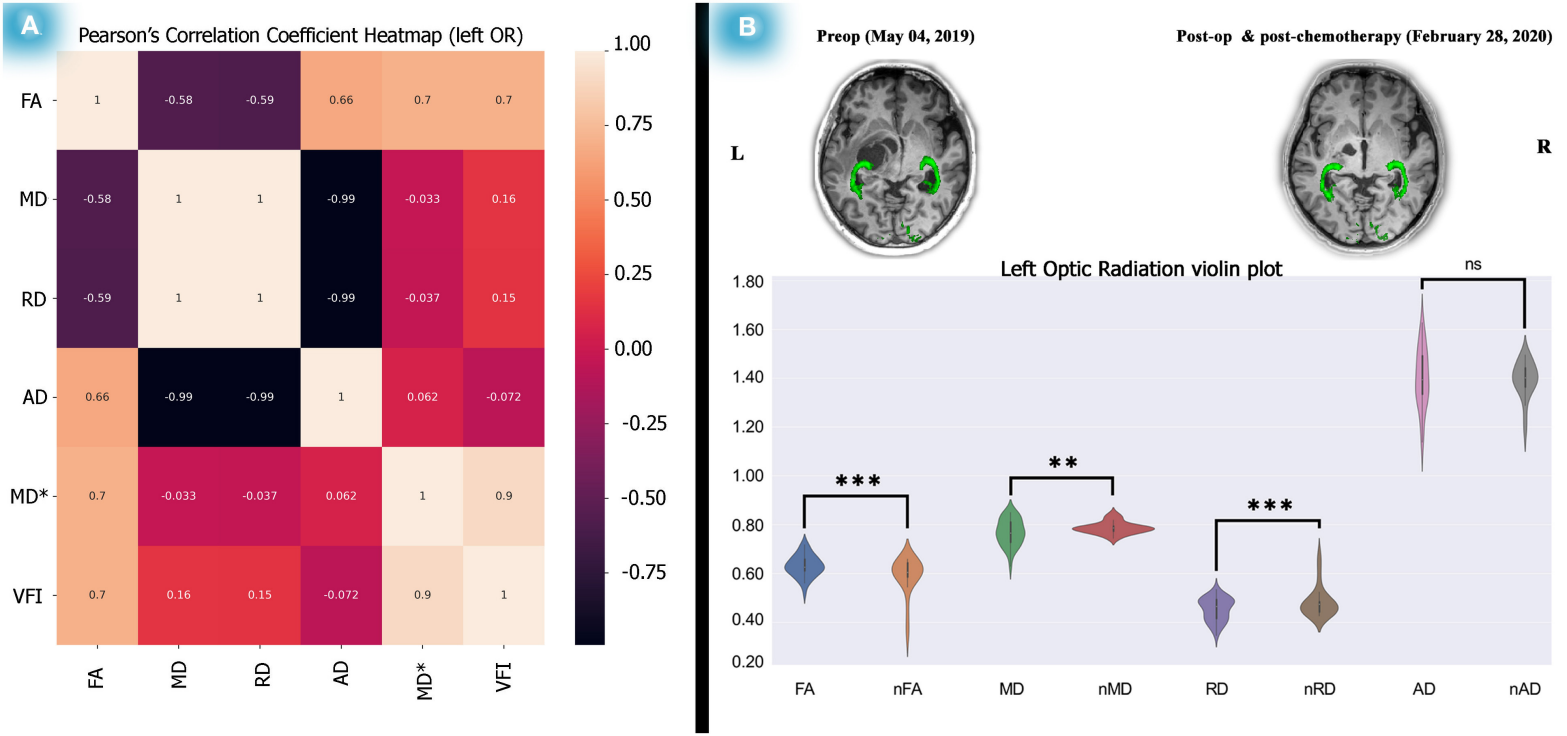
Correlation test between the left OR diffusion metrics and perimetric parameters and diffusion metric alterations after 1 year. Analyses on the relationship between the left OR and VFI showed a significant correlation between left OR FA and the left VFI presented as a heatmap matrix **(A)**. Statistical analyses on diffusion metrics of post-chemotherapy vs. normal control subjects as illustrated by violin plots revealed increased FA, decreased MD and RD, a picture of myelination, and dense axonal packing in the left OR **(B)**. The relationship analyses done by the Pearson's correlation coefficient test and the statistics performed on the diffusion metrics by two-tailed sample *t*-test, *P < 0.05; **P < 0.01; ***P < 0.001; ****P < 0.0001.

To trace the diffusion changes over time, diffusion metric (FA, MD, RD, and AD) values were extracted; a two-tailed sample *t*-test was done between the patient's and normal subjects' diffusion metrics at each stage, and the results were displayed as violin plots (see Figures 3, 4; Supplementary Figure 3). All statistical analyses were done using Python 3.7.3 (Rossum and Drake, 2014).

#### Spearman's and Pearson's Correlation Coefficient Tests

Spearman's correlation coefficient test was performed to determine the possible association between DTI metrics and perimetric parameters. We chose Spearman's correlation test because it does not keep any assumptions about distributing the data. It is the most appropriate correlation analysis when the variables are measured on a scale that is at least ordinal, which is true of our dataset.

Moreover, we used a Python data visualization library (based on Matplotlib) to visualize significant perimetric parameter changes among dMRI metrics. Doing this allowed us to create a Pearson's correlation coefficient matrix heatmap, a two-dimensional graphical data representation wherein the matrix values are represented as colors (Bedre, 2020) and used to compute pairwise correlations of columns (excluding null values).

## Results

### Immunohistochemistry (IHC) Examinations

According to the IHC results (see Supplementary Figure 4) and based on previous studies, significant attributes of the PA were revealed in which case bright red corkscrew-shaped Rosenthal fibers (RFs) are often found in compact regions (A), as they are biphasic with alternating compact and microcystic areas displayed (B). The existence of Mulberry-shaped eosinophilic granular bodies (EGBs) is also regular in the loose fraction (C) (Collins et al., 2015; Perry and Wesseling, 2016).

### Perimetry Analyses

We examined the SWAP for visual field evaluation overtime after surgery and chemotherapy. As shown in Figure 2A, pre-operation SWAP examination showed the complete involvement of both visual fields due to the left hemisphere PA and its massive edema with substantial pressure on the left OPs.

After 6 months of follow-up, right homonymous hemianopia (RHH) displayed as predicted due to the PA pressure on the left side geniculate WM tracts. Based on our hypothesis, treatment was maintained by chemotherapy, and SWAP examinations remained in place for post-chemotherapy until March 2020. Visual Field Index (VFI) and Mean Deviation (MD) properties were compared over time using the polynomial regression method.

Our longitudinal polynomial regression on the left VFI (see Figure 2B) showed a significant improvement (third-degree *R*^2^ = 0.9926, *P* < 0.001) as well as left MD, which increased over time as revealed by the third-degree polynomial regression analysis (third-degree *R*^2^ = 0.9775, *P* < 0.01).

Additionally, the same method was also used for the right visual field (see Figure 2C); in this case, the third-degree longitudinal polynomial regression on VFI was significant (third-degree *R*^2^ = 0.9979, *P* < 0.001). Moreover, the right side MD showed a considerable increase over time, and the (third-degree *R*^2^ = 0.9973, *P* < 0.001).

As described, we calculated Spearman's rank correlation coefficients between dMRI metrics and perimetric parameters. We found only a significant association (see Supplementary Tables 2, 3) between the left OR's FA and the left VFI along time (*r*_s_ = 0.976, *P* (2-tailed) < 0.001). Interestingly, Pearson's correlation analysis also showed a significant association (see Figure 4A) between the left side OR's FA and the VFI (*R*^2^ = 0.7011, *P* = 0.0025). However, no association was found in the right OR's diffusion metrics and perimetric parameters (see Supplementary Tables 2, 3; Supplementary Figure 5).

### Differential Tractography and Statistical Findings

#### Three Months Post-operation vs. Pre-operation

Using differential tractography, left OR showed 0 to 15 % increased connectivity in 3 months after surgery and a two-tailed sample *t*-test showed significant FA reduction (*t* = −5.23, *P* < 0.00001), increased MD (*t* = 3.75, *P* < 0.0001), RD (*t* = 2.42, *P* = 0.017), and AD (*t* = 2.13, *P* = 0.033) relative to normal controls, which could be a reflection of cerebrospinal fluid (CSF) adjoining to the left OR (see Supplementary Figure 3A; Supplementary Table 4A). Additionally, no significant changes in the right OR's diffusion metrics were observed in the statistical analyses.

#### Post-chemotherapy Relative to Three Months Post-operation

The same method was used for post-chemotherapy versus post-operation, shown in Supplementary Figure 3B. Our longitudinal differential tractography showed a 10–30% increased connectivity in the left and right ORs, and our statistical analyses on the left OR's diffusion metrics also showed no significant alteration of FA. However, a significant decrease was observed in MD (*t* = −3.39, *P* < 0.01) and RD (*t* = −4.451, *P* < 0.0001), and no significant changes were observed in the left OR's AD.

The statistical analyses on the right OR showed no significant diffusion metrics changes except for MD (*t* = 3.12, *P* < 0.01) in which depletion was observed. However, following the lack of changes in the other diffusion metrics, no pathological reflection could be considered for both sides of the ORs (see Supplementary Table 4B).

#### Pre-operation in Comparison With Pre-chemotherapy

The individual connectometry (see Figure 3A) showed 10–30% and 10–20% increases in connectivity in the left and right ORs.

Interestingly, our statistical analyses (see Figure 3A; Supplementary Table 4C) showed fiber maturation in the left OR in which case a significant increase in FA (*t* = 4.13, *P* < 0.001) and AD (*t* = 2.195, *P* < 0.05) was observed. Moreover, MD (*t* = −3.62, *P* < 001) and RD (*t* = −4.59, *P* < 0.0001) showed a significant decrease. Also, no meaningful changes were seen in the right OR's diffusion metrics (see Supplementary Table 4C) except for in MD (*t* = −2.428, *P* < 05), which would not reflect any pathological properties.

### Post-chemotherapy Relative to Pre-chemotherapy

Figure 3B shows increased connectivity in the left (10–30%) and right (0–10%) ORs. Our two-tailed sample *t*-test displayed significant MD reduction (*t* = −4.403, *P* < 0.0001) and RD (*t* = −2.242, *P* < 0.05) in the left OR. Moreover, a significantly increased MD (*t* = 2.49, *P* < 0.05) on the right OR was observed. Subsequently, in this period (10 months after operation), no remarkable pathological variation was detected in both ORs according to our statistical analyses (see Supplementary Table 4D).

### Post-chemotherapy vs. Pre-operation

Concerning evaluating the changes in the OR's diffusion metrics over time, the differential tractography approach was used to measure our treatment strategy outcome. We decided to compare the pre-operation and post-chemotherapy diffusion properties (see Figure 3C). This comparison indicated crucial changes during 10 months of DTI follow-up.

Our statistical analyses which are presented as the violin plots demonstrated a significant increase in FA (*t* = 2.63, *P* = 0.01), remarkable decreased MD (*t* = −2.98, *P* < 0.01), RD (*t* = −2.24, *P* < 0.05), and a non-significant change in AD (see Supplementary Table 4E). These findings could indicate myelination and dense axonal packing of the left OR. Also, no diffusion metric changes were observed in the right OR.

### Whole-Brain Differential Tractography: Connectivity Alterations Over One Year

Our study focused on using ROI-based differential tractography to illustrate conventional chemotherapy's efficacy for the NF1-OPG. To this end (and to better understand the treatment strategy), we used a non-specific region-based differential tractography approach. This process provides a picture of the whole-brain WM connectivity alterations over 1 year.

We created a connectometry database from post-operation DTI scans and used the individual connectometry approach. This method allowed us to compare pre-operation and post-operation whole-brain WM integrities over time. As shown in Supplementary Figure 6, the left and right cingulum's connectivity, genu, and body of the corpus callosum increased by 10–30% over 10 months.

## Discussion

In the present study, a 25-year-old female was diagnosed with NF1 adjacent to a left hemisphere glioma with massive edema for which the left OR was under high pressure. Based on the clinical examinations and SWAP test, limited visual acuity was an obvious consequence.

To eliminate the pressure of the glioma on the OPs, the patient underwent surgery, and, based on our hypothesis, to prevent adverse effects like OPs injury, neurocognitive dysfunctions, and cerebrovascular complications, the post-operation treatment was done by chemotherapy rather than radiation. Differential tractography adjoining SWAP tests were done for 1 year following surgery to evaluate the visual field and DTI metrics alterations of the OPs to evaluate this treatment strategy.

In addition to our experience with NF1 patients, PA was also revealed by IHC analyses, and the study was designed based on neuroimaging data and SWAP examinations. This information was obtained longitudinally four times every 3 months, and the VFI changes were demonstrated over time using the polynomial regression method.

On the other hand, differential tractography was used to evaluate the diffusion metrics of the OPs over time. This procedure was divided into five parts. Individual connectometry was performed to find out the possibility that the OP's diffusion would increase over time. Also, statistical analyses illustrated the longitudinal diffusion alterations relative to normal subjects.

The ORs showed diffusion alterations during the post-operation treatment, reflecting myelination dense axonal packing (see Figure 4; Supplementary Table 4E). Also, the SWAP examinations revealed significant improvements over time (see Figure 2). Moreover, the association between the left side OR's FA and the left VFI was substantial, as indicated by Spearman's and Pearson's correlation analyses (see Figure 4A, Supplementary Tables 2, 3).

Based on our hypothesis that post-operation chemotherapy could be more effective than radiation therapy for preventing OPs injury, ROI-based differential tractography and SWAP examinations adjacent to statistical analyses on diffusion metrics were done over ten months. To the best of our knowledge, this study was the first to use these approaches.

### Optic Pilocytic Astrocytoma (PA) and Neurofibromatosis Type 1 (NF1)

OPGs affect 15-20% of NF1 patients, and they are not usually biopsied. Consequently, the most common tumors in NF1 patients are of the glioma type. Further, most of the NF1-OPGs are categorized as PAs (Lewis et al., 1984; Listernick and Charrow, 1990; Campen and Gutmann, 2018).

PAs can occur in different brain regions and are frequently found within the OP and the brainstem. About 66% of gliomas are located in the OP and the brainstem in NF1 patients (Guillamo et al., 2002).

Our case was a young adult NF1 patient with a PA located on the left white matter OR. Following the imaging details, the OR's glioma was observable, and no biopsy was needed. The post-operation IHC analyses revealed OR-PA.

According to the literature, low-grade OPGs are not confounding, and the post-operation treatment strategy was the main point of our study. Hence, chemotherapy (rather than radiation) was selected for the post-operation treatment to prevent OR injury, cognitive disorders, and cerebrovascular complications which the treatment strategy was examined via longitudinal differential tractography and SWAP tests.

### SWAP Feasibility in an NF1 Patient With Optic Radiation Glioma

Several perimetric tests can be applied to an individual patient with the glaucomatous progression that they might detect, such as short-wavelength automated perimetry (SWAP) (Drance et al., 1981; Flammer and Drance, 1984), standard automated perimetry (SAP) (Anderson and Patella, 1999), and frequency-doubling technology (FDT) perimetry (Johnson et al., 1999). A comparative study on glaucoma progression utilized these methods and observed no statistically significant difference between SAP, SWAP, and FDT via the pointwise linear regression (PLR) method (Hu et al., 2016).

Previously, SAP, FDT, and SWAP were compared to identify glaucomatous visual loss utilizing the Swedish interactive thresholding algorithm. However, no significant differences were found in their diagnostic performance (Tafreshi et al., 2009).

New special diagnostic instruments such as optical coherence tomography (OCT) and ganglion cell layer–inner plexiform layer (GCL-IPL) thickness have provided new FOV that can be used to investigate the ophthalmic indications of NF1 patients with optic nerve glioma (Gu et al., 2014; Abdolrahimzadeh et al., 2016). As previously described, our case is a young woman with optic radiation PA, not optic nerve glioma. Subsequently, as recommended by previous investigations, we decided to use SWAP to make longitudinal observations of her visual field.

Previous investigations revealed no differences between the abilities of SAP, SWAP, and FDT to monitor glaucomatous progression. However, in a study based on PLR analyses, FDT sensitivity was detected faster than SAP and SWAP in a cohort of glaucoma patients (Liu et al., 2014). Such comparative studies are ongoing. Nevertheless, SWAP could be disadvantageous for observing our patient's perimetry alterations over time.

Meanwhile, in a recent study on perimetric anomalies, the researchers utilized FDT instead of SWAP in NF1 patients. A significant alteration was found in the transmission of visual impulse and FDT analyses, representing a significant reduction in all observed parameters, including (central sensitivity (CS), mean deviation (MD), pattern standard deviation (PSD), and glaucoma hemifield test (GHT). These findings point to the involvement of the visual pathway (Nebbioso et al., 2020).

Optic neuropathy is characterized by damaged optic pathway axons, which could cause retinal ganglion cell (RGC) death. The primary damage areas are the optic nerve head (ONH) and the lateral geniculate pathways (Vrabec and Levin, 2007). This is relevant to our study, as our case is an NF1 patient with left geniculate pathway PA.

SWAP is designed to detect visual losses caused by optic neuropathy. In this perimetry method, short-wavelength sensitive (sws) cones are used, as humans' vision is most susceptible to this wave-length. These sws signals are sent to the blue-yellow retinal ganglion cells and will detect the target at any given retinal location when the blue-yellow cells are damaged (Sample, 2000).

Based on the neurobiology of RGCs due to the damaged OPs and the power of SWAP to detect the RGCs, to the best of our knowledge, we had no longitudinal visual function monitoring research via SWAP in NF1 OPG patients unexpectedly. Several studies proved that no significant difference was observed between SAP, SWAP and FDT using different statistical methods.

### Radiotherapy or Chemotherapy for NF1 Patients Associated With OPGs

A previous multi-center investigation (Guillamo et al., 2002) of 104 NF1 patients with OP tumors reported that among 28 patients who underwent radiotherapy, 13 (46%) experienced growth hormone shortcomings. Another nine patients (32%) experienced radiation-related ischaemic strokes, and six patients (21%) underwent no radiotherapy.

According to this investigation, chemotherapy is a suitable alternative to radiotherapy. However, a few published papers (Listernick et al., 1999) suggest that NF1 patients with tumors that cause neurological symptoms may require surgical resection or chemotherapy (Gutmann et al., 2017).

More recently, treatments have become more focused on vision maintenance. This was initiated from a multi-center study, wherein carboplatin and vincristine as chemotherapeutic agents were applied instead of radiation on OPGs for initial therapy (Packer et al., 1997).

Procarbazine, lomustine, and vincristine increase survival in LGGs, but they correlate with major hematologic, hepatic, neurologic, and cutaneous toxicity (Jutras et al., 2018).

Following our study and previous investigations, radiation therapy is not useful for OPGs, particularly for NF1 tumor patients. NF1 patients commonly have PA as an LGG tumor, and radiotherapy could increase the risk of vasculopathy complications and incidental malignancies (Grill et al., 1999; Sharif et al., 2006).

Single-agent TMZ and multi-agent PCV are both listed as equally appropriate options (Ziu et al., 2015), and TMZ and PCV provide equivalent objective response rates (45–62%) with a toxicity profile favoring TMZ in terms of better tolerability. Nevertheless, we used TMZ for chemotherapy in our case—the outcome was satisfactory.

Curiously, the authors of one previous study concluded that radiation therapy is an effective treatment for optic LGG and that older children without NF-1 have a low risk of late toxicity (Tsang et al., 2017).

Our analyses, considered alongside previous findings, suggest that chemotherapy is the best post-operation therapy technique for low-grade OPGs. It must be considered that our investigation is a longitudinal clinical case study of a single NF1-OPG patient, and, thus, the power of generalization is limited.

### Longitudinal Diffusion MRI Adjoining Differential Statistics

After reviewing the literature (and according to the association between white matter optic PA tumors and NF1 patients), we decided to focus on the ROI-based differential tractography analyses. Doing this enabled us to trace the connectivity changes of the OPs over time.

In addition to the statistical analyses, a feasible association picture of dMRI and perimetric parameters were obtained. Significant Pearson's correlation between left OR's FA and the left VFI (R^2^ = 0.7011, P-Value = 0.0025), illustrated as a heatmap matrix (see Figure 4A). The Spearman's correlation (see Supplementary Table 2) also showed the signification (r_s_ = 0.976, P (2-tailed) <0.001).

However, the Spearman's and Pearson's correlation analyses failed to detect a significant association between dMRI and perimetric parameters of the right side OR and VFI by methods (see Supplementary Table 2; Supplementary Figure 5). Likewise, our statistical analyses of mean deviation and diffusion alterations utilizing the mentioned statistical analysis methods showed no significant association (see Supplementary Table 3).

Several investigations on OPGs using dMRI revealed relationships between the dMRI and perimetric parameters (Ciccarelli et al., 2005; de Blank et al., 2013, 2018; Kolbe et al., 2016), thus supporting our results. The association between FA and visual acuity parameters is commonly observed in these papers, further supporting our findings.

However, some investigations utilizing dMRI were done previously on NF1-OPGs. However, to the best of our knowledge, the longitudinal ROI-based differential tractography method has not been investigated. Therefore, this approach was applied in this study to analyze diffusion changes of the ORs over time.

In an investigation using diffusion MRI (Hales et al., 2018), fractional anisotropy (FA) and apparent diffusion coefficient (ADC) found that the values of OPs were correlated with visual acuity in 26 patients. However, no longitudinal analyses were performed, and only FA and ADC values were extracted for analyses. Interestingly, according to their results, lower FA was associated with poorer vision, which is in line with our study results (see Figure 4A; Supplementary Table 3). In addition, based on our findings, increased FA (*t* = 4.43, *P* < 0.0001) and decreased MD (*t* = −3.81, *P* < 0.001) and RD (*t* = −4.56, *P* < 0.0001) showed possible myelination and dense axonal packing in the left OR (see Figure 4B; Supplementary Table 4E) after 1 year.

Similar results were also reported in a previous investigation (de Blank et al., 2013). Specifically, the initial FA of ORs was associated with visual acuity in a 1-year follow-up. In contrast, in our study, diffusion MRI was assessed four times every 3 months alongside SWAP examinations. However, this study was done on 50 children with NF1-associated OPGs, and our study involved one young adult NF1-OPG case.

Another study that employed tract-based spatial statistics (Smith et al., 2006) on 40 children with NF1 claimed that reduced FA values were correlated with multiple WM regions (Zamboni et al., 2007; Karlsgodt et al., 2012; de Blank et al., 2020). The study's main limitation was that it is done on developing brains, whereas our investigation was longitudinal on a rare young adult woman with NF1-OPG.

In our study, the dMRI assessment after chemotherapy in the 1-year follow-up showed increased FA and decreased MD and RD values in the left OR (see Figure 4, Supplementary Table 4E). However, in the mentioned study (de Blank et al., 2020), decreased FA and increased MD and RD values were reported in nearly all WM regions.

However, these results were reported in terms of WM maturation, which is in contrast to the previous studies regarding the relationships between diffusion metrics and brain pathology (Feldman et al., 2010; Alexander et al., 2011); these demonstrated these changes as a form of axonal degeneration, which would be more reliable for NF1 properties.

As illustrated in Figure 4, post-chemotherapy, compared with normal subjects, revealed significant FA increases and MD and AD reductions (see Supplementary Table 4E) related to axonal packing and myelination. This supports the hypothesis that NF1 associated with OPGs does not require radiation therapy, as such therapy could induce cognitive problems, cerebrovascular complications, and OPs injury.

## Limitations of the Study

The present study is based on a dataset collected from a single participant, and thus, its generalizability is limited. Moreover, to reach a purpose-based criterion for glioma precision therapy, managing genetic, cellular, and behavior imaging in associated-NF1 patients with OPGs is a crucial step toward precision tailored treatment.

Also, deterministic tractography used in every step of treatment fits the WM pathway step by step, using diffusion directions in seed voxels to determine the next voxel in the tract and rely on minimum FA and maximum turning angle to reduce inaccurate tract directions. However, these procedures might increase the FA, which could be a disadvantage of applying this technique. However, differential tractography would be a relevant approach for evaluating visual acuity over time. It could also be a useful complement to longitudinal perimetric tests.

## Conclusions

We decided to examine an old hypothesis that insists that chemotherapy is an effective treatment for NF1 patients associated with OPGs via the longitudinal differential tractography approach and SWAP tests, adjoining differential statistical analyses. This approach allowed us to trace the ORs' diffusion changes over time and check the possible association between diffusion and SWAP parameters (see Figure 4A; Supplementary Tables 2–4).

We also used whole-brain differential tractography to determine other altered WM tracts anisotropy over time. Doing this gave us a more detailed picture of the treatment efficacy. Interestingly, the connectivity between the body and genu of the corpus callosum increased by 10–30% over 1 year (see Supplementary Figure 6). This extends our previous study on glioma invasion, which revealed that the corpus callosum body played a role in this procedure (Sharifi et al., 2019).

Future works should design algorithms from field theory, anatomical modeling, and population genetics. Such algorithms could detect a genetic continuum wherein the brain structure is heavily genetically determined in some parts but not others (Thompson et al., 2001, 2002; Winkler et al., 2010; van der Lee et al., 2019). Thus, future investigations should focus on genetic-based imaging with a large sample size in conjunction with robust, distinctive statistical analyses.

Furthermore, neuroimaging meta-analyses would be suitable for investigators who wish to obtain an appropriate FOV for this specific problem. There is plenty of meta- and mega-analyses (Hoogman et al., 2017; Mufford et al., 2017; Thompson et al., 2017; Rinker et al., 2018; Schmaal et al., 2020; Tozzi et al., 2020) that have been utilized as a prototype to evaluate a new design to enhance precision tailored therapy. Meta- and mega-analyses could provide a proper FOV for pathological genomic imaging (Thompson et al., 2010) and basic science investigators in the future.

## Data Availability Statement

The original contributions presented in the study are included in the article/Supplementary Material, further inquiries can be directed to the corresponding author/s.

## Ethics Statement

Written informed consent was obtained from the individual(s), and minor(s)' legal guardian/next of kin, for the publication of any potentially identifiable images, or data included in this article.

## Author Contributions

AP designed the study, acquired, analyzed, and explained the data, wrote the manuscript, and revised it. GS and MS are the main neurosurgeons of the case. AA is the main neuro-oncologist of the study. FB-Z and AP performed IHC analyses. SN and TM revised the manuscript. All authors contributed to the article and approved the submitted version.

## Conflict of Interest

The authors declare that the research was conducted in the absence of any commercial or financial relationships that could be construed as a potential conflict of interest.

## Footnotes

^1^NCT00003466, N. N. Temozolomide in Treating Patients With Progressive Low-Grade Glioma, https://ClinicalTrials.gov/show/NCT00003466.

## Supplementary Material

Supplementary Figure 1 The Clinical manifestations of the NF1 young adult woman. Café au lait spots and neurofibromas on her front and back body **(A,B)**. Freckling in the axillary regions **(C)**. An OP glioma on the left geniculate WM. NF1, neurofibromatosis type 1; OP, optic pathway; WM, white matter.

Supplementary Figure 2 Differential tractography according to the FDR protocol (Yeh et al., 2019). Three baseline scans were used to differentiate the pre-operation, pre-chemotherapy, and post-chemotherapy vs. SHAM scan wherein the ORs are displayed by red color. FDR, false discovery rate; OR, optic radiation.

Supplementary Figure 3 Differential tractography approach and statistical analyses to discover the ORs connectivity and diffusion metric alterations. Increased connectivity of the left OR in 3 months post-operation vs. pre-operation. Statistical analyses revealed FA reduction, increased MD, RD, and AD relative to nFA, nMD, nRD, and nAD, respectively **(A)**. The method utilized for post-chemotherapy vs. post-operation and presented increased connectivity of the left OR. The violin plots of diffusion metrics showed no significant pathological reflection **(B)**. Statistics performed on the diffusion metrics by two-tailed sample *t*-test, ^*^*P* < 0.05; ^**^*P* < 0.01; ^***^*P* < 0.001; ^****^*P* < 0.0001. OR, optic radiation; FA, fractional anisotropy; MD, mean diffusivity; RD, radial diffusivity; AD, axial diffusivity; nFA, normal FA; nMD, normal MD; nRD, normal RD; nAD, normal AD.

Supplementary Figure 4 The IHC analyses recognized the OP tumor as PA. Bright red, corkscrew-shaped Rosenthal fibers (RFs) are often found in compact regions **(A)**. PAs are mostly biphasic with alternating compact and loose/microcytic growth patterns **(B)**. Mulberry-shaped eosinophilic granular bodies (EGBs) are most common in the loose component **(C)**. IHC, Immunohistochemistry; OP, optic pathway; PA, pilocytic astrocytoma.

Supplementary Figure 5 Correlation analyses performed between the right OR diffusion metrics and perimetric parameters. Diffusion metrics of the right OR and perimetric parameters (VFI and MD) were extracted. The correlation analyses were performed by Pearson's correlation coefficient test and illustrated as a matrix heatmap. OR, optic radiation; VFI, visual field index; MD, mean deviation.

Supplementary Figure 6 Whole-brain differential tractography performed to identify the other WM tracts connectivity alteration over time. QA changes traced with utilizing three different thresholds (+10%, +20%, and +30%) to make connectivity comparisons between time points. Genu and body of the CC and left, and right cingulum showed increased connectivity over time. WM, white matter; QA, quantitative anisotropy; CC, corpus callosum.

Supplementary Table 1 The FDR correction protocol was done five times to differentiate 3 months post-operation vs. pre-operation, post-chemotherapy relative to 3 months post-operation, pre-operation vs. pre-chemotherapy, post-chemotherapy vs. pre-chemotherapy, and post-chemotherapy relative to pre-operation. The FDR = (total number of differential tracks count from sham scans) / (total number of differential tracks count from the follow-up scans), ^*^*P* < 0.05. FDR, false discovery rate.

Supplementary Table 2 Spearman's correlation coefficient test performed between diffusion metrics and VFI. The correlation analyses were performed between each dMRI metric and VFI at each time point. A significant association between left OR FA and the left VFI was found over time, ^***^*P* < 0.001. VFI, visual field index; dMRI, diffusion magnetic resonance imaging; OR, optic radiation; FA, fractional anisotropy.

Supplementary Table 3 Spearman's correlation coefficient test performed between diffusion metrics and MD. The correlation analyses were performed between each dMRI metric and MD at each time point. No significant association between each side of OR diffusion metrics and the MD was found over time. MD, mean deviation; dMRI, diffusion magnetic resonance imaging; OR, optic radiation.

Supplementary Table 4 Statistical analyses between patient and normal ORs dMRI metrics performed in five stages including 3 months post-operation vs. pre-operation **(A)**, post-chemotherapy relative to 3 months post-operation **(B)**, pre-operation vs. pre-chemotherapy **(C)**, post-chemotherapy vs. pre-chemotherapy **(D)**, and post-chemotherapy relative to pre-operation **(E)**. Statistics performed by two-tailed sample *t*-test, ^*^*P* < 0.05; ^**^*P* < 0.01; ^***^*P* < 0.001; ^****^*P* < 0.0001. OR, optic radiation; dMRI, diffusion magnetic resonance imaging.

## Abbreviations

DTI: Diffusion Tensor Imaging
MRI: Magnetic Resonance Imaging
dMRI: diffusion MRI
IHC: Immunohistochemistry
NF1: Neurofibromatosis type 1
OP: Optic Pathway
OPG: Optic Pathway Glioma
LGG: Low-Grade Glioma
PA: Pilocytic Astrocytoma
SWAP: Short-Wavelength Automated Perimetry
OR: Optic Radiation
WM: White Matter
TMZ: Temozolomide
FDR: False Discovery Rate
FA: Fractional Anisotropy
MD: Mean Diffusivity
RD: Radial Diffusivity
AD: Axial Diffusivity
ROI: Region Of Interest
VFI: Visual Field Index
MD: Mean Deviation
RGCs: Retinal Ganglion Cells Death
PCV, Procarbazine: CCNU and Vincristine.
