# Supplementary figures and images for: Case Report: Chemotherapy Indication in a Case of Neurofibromatosis Type 1 Presenting Optic Pathway Glioma: A One-Year Clinical Case Study Using Differential Tractography Approach
Source: Front Hum Neurosci. 2021 Apr 6;15:620439. doi: 10.3389/fnhum.2021.620439 (PMC8115022; doi:10.3389/fnhum.2021.620439)

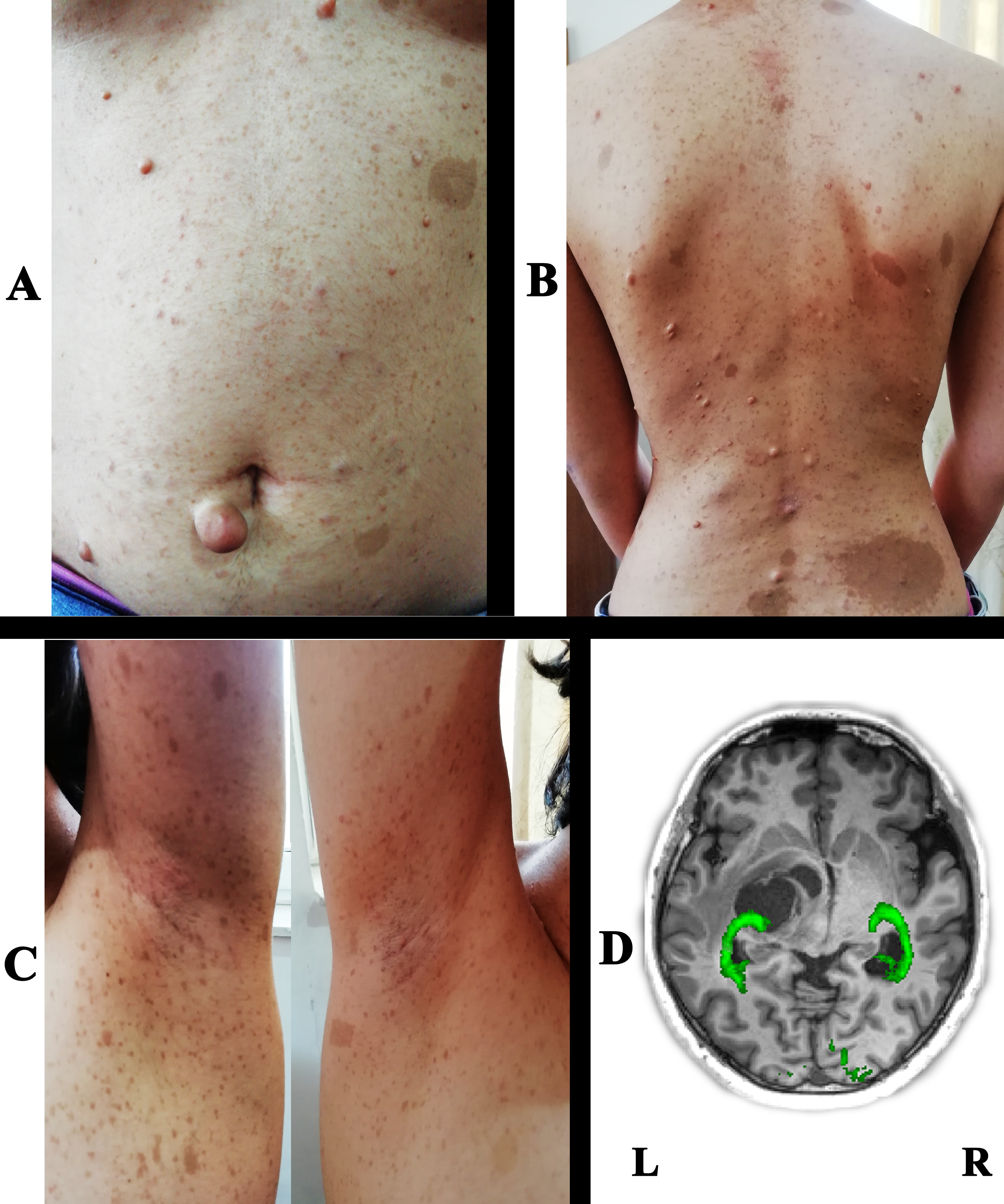

Supplement: Supplementary Figure 1 — The Clinical manifestations of the NF1 young adult woman. Café au lait spots and neurofibromas on her front and back body (A,B). Freckling in the axillary regions (C). An OP glioma on the left geniculate WM. NF1, neurofibromatosis type 1; OP, optic pathway; WM, white matter. [file Image_1.TIF]

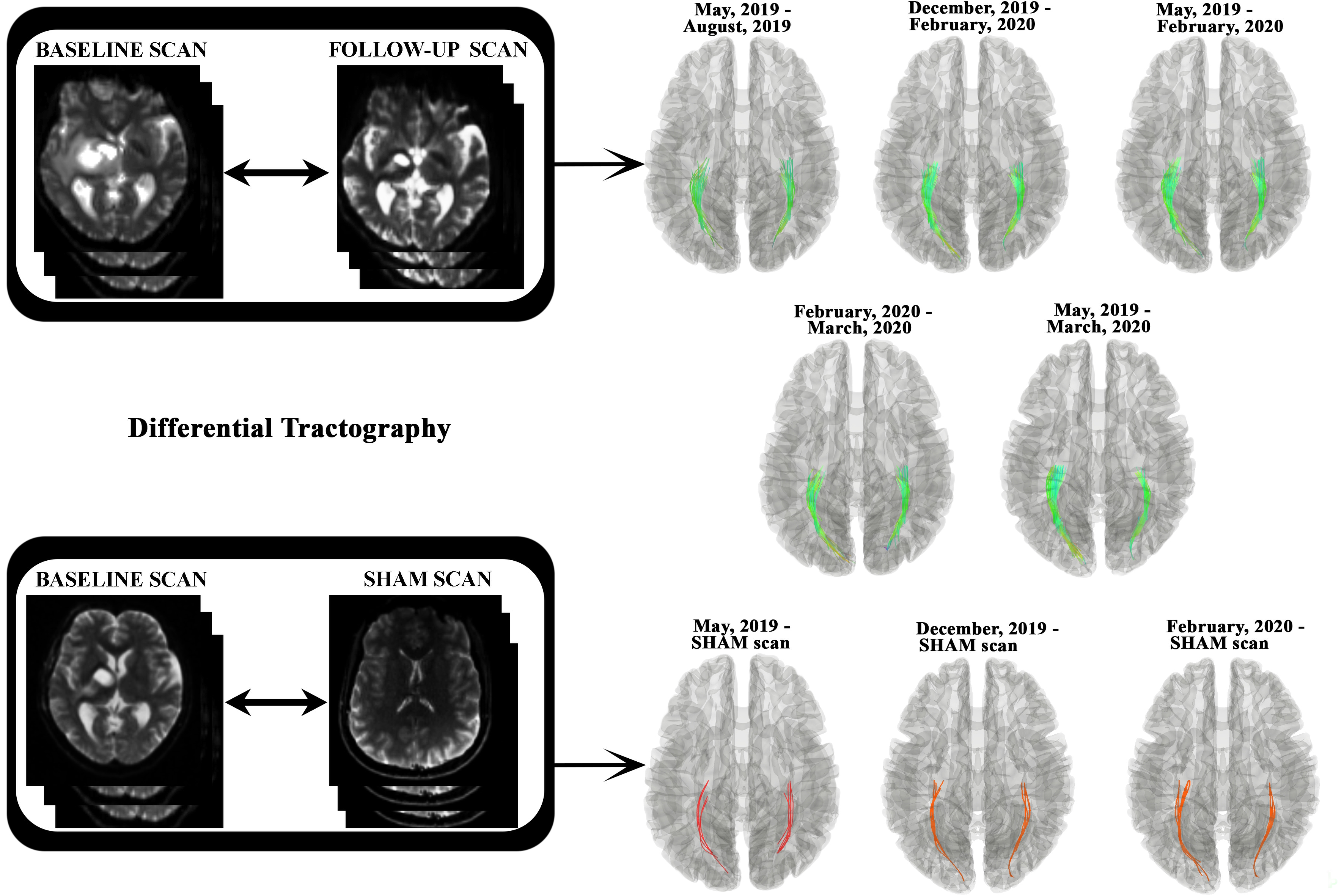

Supplement: Supplementary Figure 2 — Differential tractography according to the FDR protocol (Yeh et al., 2019). Three baseline scans were used to differentiate the pre-operation, pre-chemotherapy, and post-chemotherapy vs. SHAM scan wherein the ORs are displayed by red color. FDR, false discovery rate; OR, optic radiation. [file Image_2.TIF]

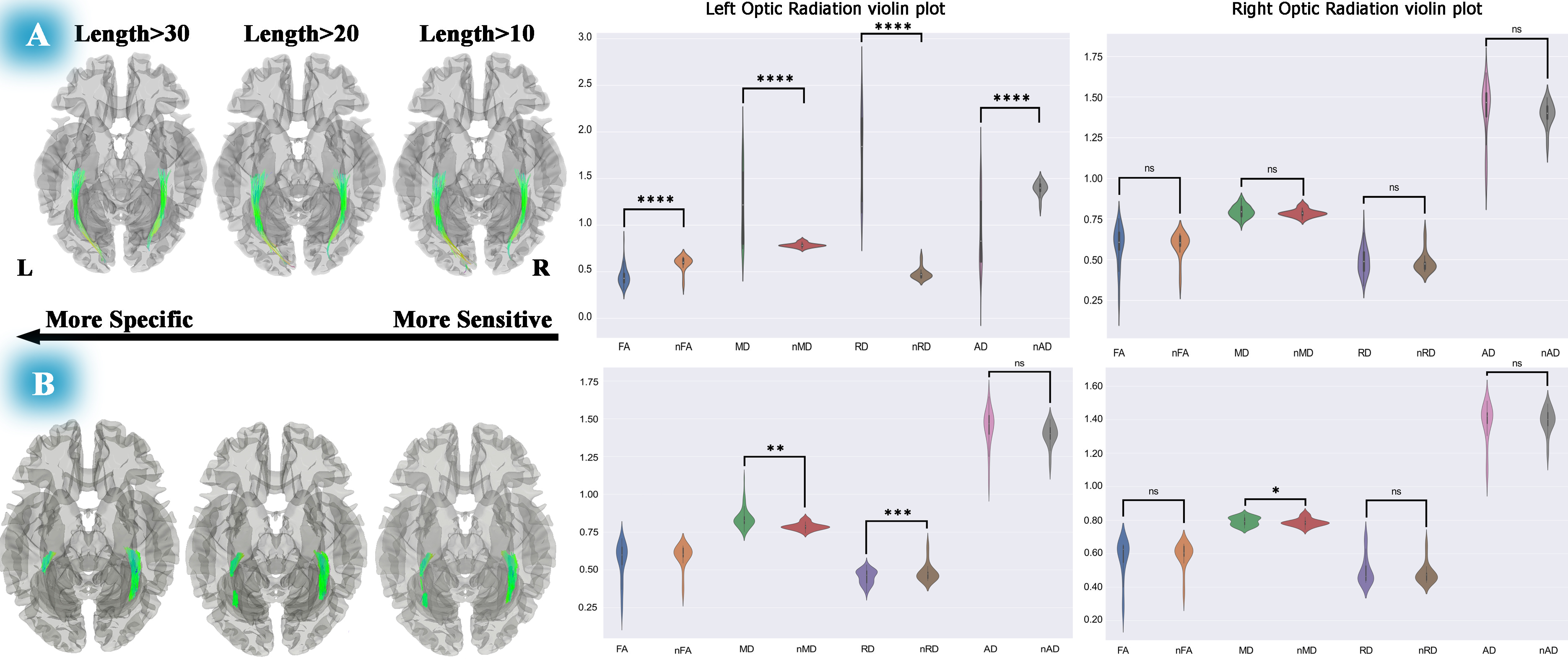

Supplement: Supplementary Figure 3 — Differential tractography approach and statistical analyses to discover the ORs connectivity and diffusion metric alterations. Increased connectivity of the left OR in 3 months post-operation vs. pre-operation. Statistical analyses revealed FA reduction, increased MD, RD, and AD relative to nFA, nMD, nRD, and nAD, respectively (A). The method utilized for post-chemotherapy vs. post-operation and presented increased connectivity of the left OR. The violin plots of diffusion metrics showed no significant pathological reflection (B). Statistics performed on the diffusion metrics by two-tailed sample t-test, *P < 0.05; **P < 0.01; ***P < 0.001; ****P < 0.0001. OR, optic radiation; FA, fractional anisotropy; MD, mean diffusivity; RD, radial diffusivity; AD, axial diffusivity; nFA, normal FA; nMD, normal MD; nRD, normal RD; nAD, normal AD. [file Image_3.TIF]

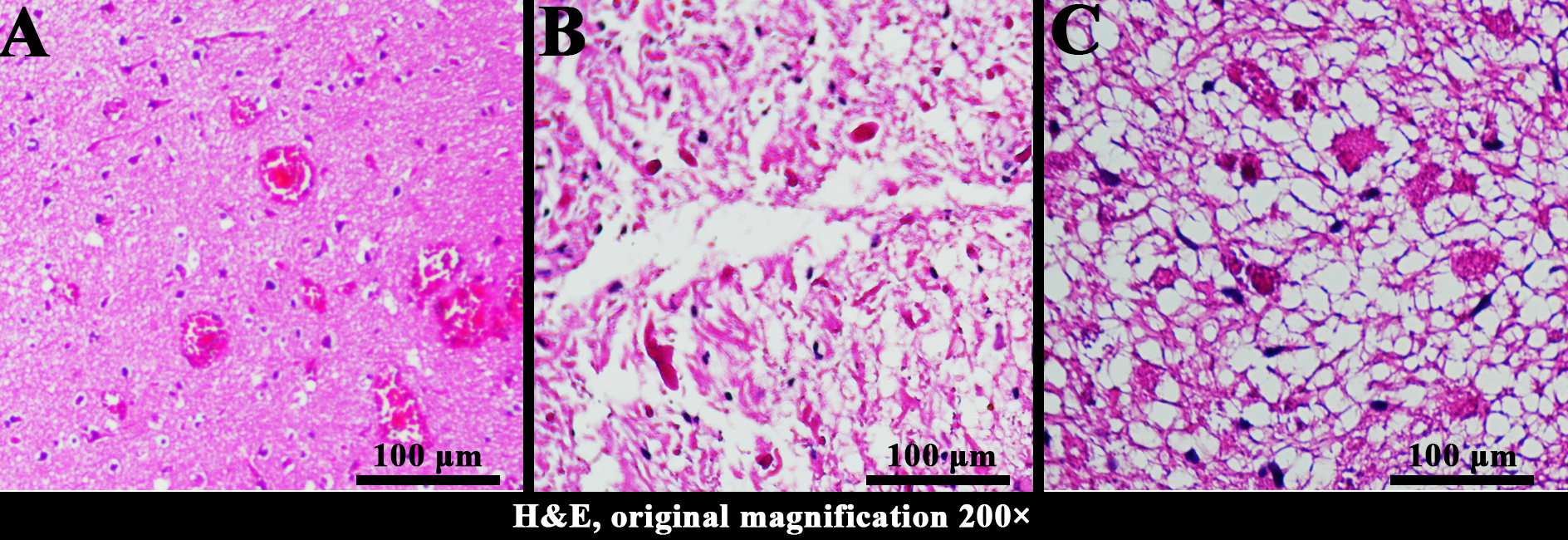

Supplement: Supplementary Figure 4 — The IHC analyses recognized the OP tumor as PA. Bright red, corkscrew-shaped Rosenthal fibers (RFs) are often found in compact regions (A). PAs are mostly biphasic with alternating compact and loose/microcytic growth patterns (B). Mulberry-shaped eosinophilic granular bodies (EGBs) are most common in the loose component (C). IHC, Immunohistochemistry; OP, optic pathway; PA, pilocytic astrocytoma. [file Image_4.TIF]

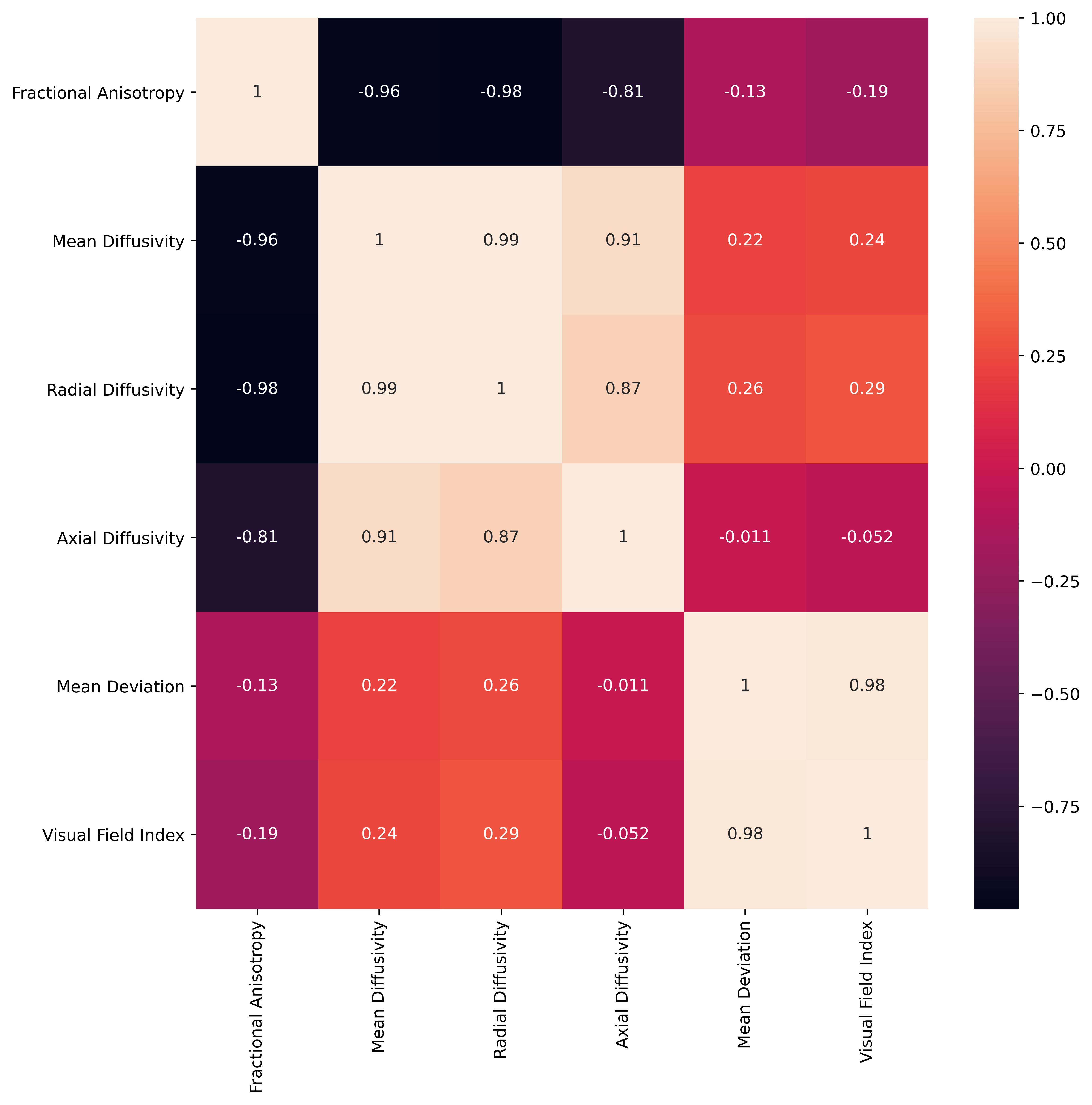

Supplement: Supplementary Figure 5 — Correlation analyses performed between the right OR diffusion metrics and perimetric parameters. Diffusion metrics of the right OR and perimetric parameters (VFI and MD) were extracted. The correlation analyses were performed by Pearson's correlation coefficient test and illustrated as a matrix heatmap. OR, optic radiation; VFI, visual field index; MD, mean deviation. [file Image_5.TIF]

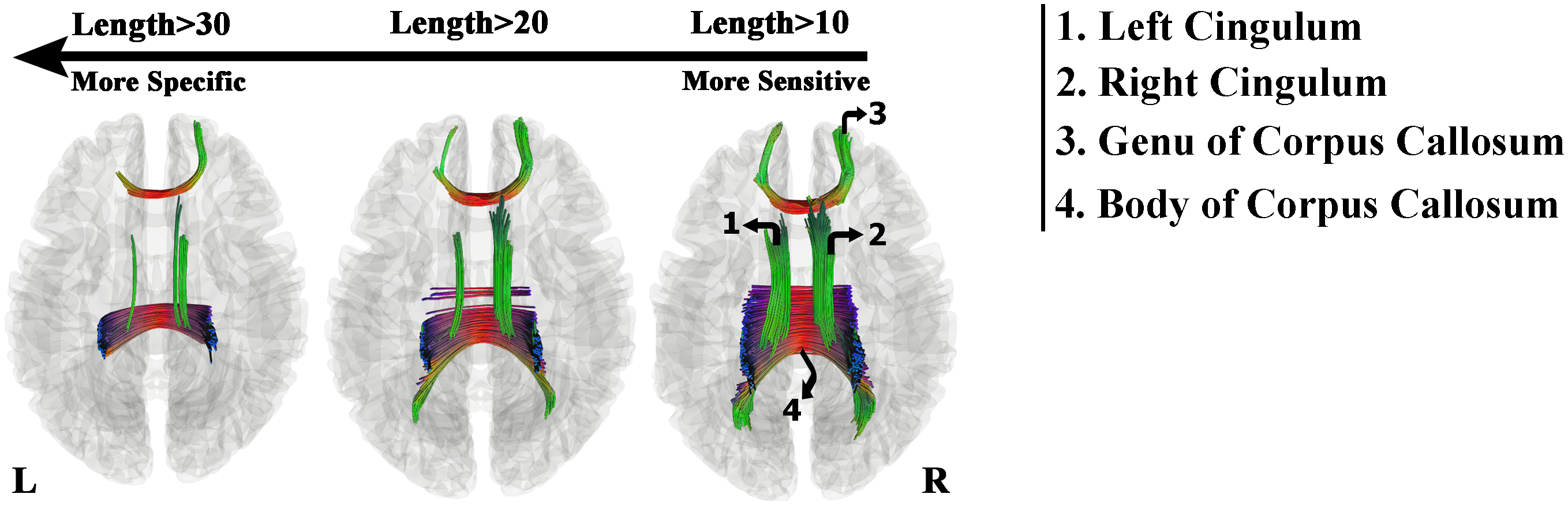

Supplement: Supplementary Figure 6 — Whole-brain differential tractography performed to identify the other WM tracts connectivity alteration over time. QA changes traced with utilizing three different thresholds (+10%, +20%, and +30%) to make connectivity comparisons between time points. Genu and body of the CC and left, and right cingulum showed increased connectivity over time. WM, white matter; QA, quantitative anisotropy; CC, corpus callosum. [file Image_6.TIF]
